# Silver nitrate functionalized rice husk-derived graphene oxide as a nanocarrier for pH-responsive drug delivery

**DOI:** 10.1186/s11671-025-04400-w

**Published:** 2025-12-20

**Authors:** Kamal Garwal, Kundan Singh Rawat, Tanuja Arya, Satish Sati, Chetna Tewari, Mintu Pal, Veena Pande, Yong Chae Jung, Nanda Gopal Sahoo

**Affiliations:** 1 Department of Chemistry, D. S. B. Campus, Prof. Rajendra Singh Nanoscience and Nanotechnology Centre, Kumaun University, Nainital, Uttarakhand 263001 India; 2 Department of Chemistry, Graphic Era Hill University, Haldwani Campus, Nainital, Uttarakhand India; 3 RAMP Convergence Research Center, Korea Institute of Science and Technology (KIST), 92 Chudong-ro, Bongdong-eup, Wanju-gun, Jeonbuk 55324 Republic of Korea; 4 Department of Pharmacology, All India Institute of Medical Sciences (AIIMS), Bathinda, Punjab 151001 India; 5 Department of Biotechnology, Sir J. C. Bose Technical Campus, Kumaun University, Bhimtal, Nainital, Uttarakhand 263136 India

**Keywords:** Rice husk derived graphene oxide, Silver nitrate, Drug delivery, Anticancer activity, 5-Fluorouracil

## Abstract

In this study, graphene oxide (GO) derived from rice husk was functionalized with silver nitrate (AgNO_3_) through a chemical co-precipitation method, and its biomedical applications were systematically investigated. Drug release experiments revealed a pH-responsive profile, where at pH 4.0 the cumulative release reached 32.6 ± 1.6% after 24 h, compared to 12.4 ± 1.8% at pH 7.4, highlighting its potential for targeted delivery to cancer cells. Reactive oxygen species (ROS) analysis and flow cytometry demonstrated that RH-GO/AgNO_3_ treatment elevated oxidative stress and triggered apoptosis in HeLa cells. Furthermore, 5-fluorouracil (FU) was successfully loaded onto the nanocomposite surface via non-covalent interactions. Cytotoxicity assessment by MTT assay showed that FU-loaded RH-GO/AgNO_3_ had the strongest anticancer activity, with an IC_50_ value of 256.3 µg/mL. ROS levels in treated HeLacells increased significantly (40.17%) compared to the control group (19.45%), and flow cytometry confirmed a reduction in cell viability (69.5%) accompanied by enhanced apoptosis (early: 8.3%, late: 4.0%) and necrosis (18.2%). Collectively, these findings indicate that RH-GO/AgNO_3_-FU induces cancer cell death predominantly via apoptosis. Overall, this work demonstrates that agricultural waste-derived nanomaterials can serve as cost-effective and sustainable platforms for advanced drug delivery in cancer therapeutics.

**Graphical abstract:**

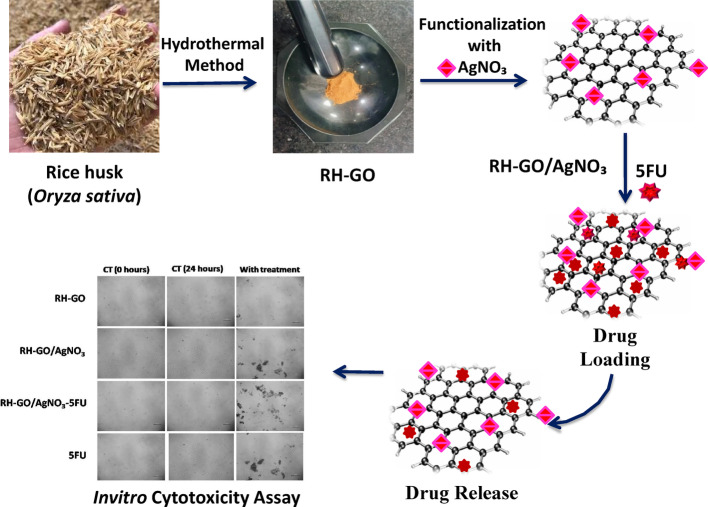

**Supplementary Information:**

The online version contains supplementary material available at 10.1186/s11671-025-04400-w.

## Highlights

Eco-friendly synthesis of RH-GO functionalized with AgNO_2_ from rice husk for biomedical applications using agricultural waste.

RH-GO/AgNO_2_ nanocomposites showed enhanced 5FU loading 16.25% and pH-responsive drug release 32.6%.

The 5FU-loaded RH-GO/AgNO_2_ exhibited strong cytotoxicity with an IC50 value of 256.3 µg/mL in HeLa cells.

ROS levels increased to 40.17% in treated cells, indicating oxidative stress-induced apoptosis.

**Supplementary Information:**

The online version contains supplementary material available at 10.1186/s11671-025-04400-w.

## Introduction

Approximately 600 million tons of rice husks (RH) are discarded annually worldwide, with most either burnt in furnaces or dumped using land-intensive methods, creating environmental concerns [1]. RH, an abundant agricultural by-product, consists of 65–75% organic matter (cellulose, hemicellulose, and lignin) and 15–20% inorganic components, mainly silica, making it a sustainable raw material for high-value-added nanomaterials [2, 3]. When combusted in an oxygen-rich atmosphere, RH produces both heat energy and silica-rich ash, which can be further utilized in diverse applications. In recent years, low-cost conversion of RH into nanostructures has attracted significant attention, as this valorization not only supports waste management but also provides economic and environmental benefits. RH-derived nanomaterials, such as graphene, carbon nanotubes, carbon dots, fullerenes, and carbon nanofibers, have demonstrated enormous potential in biomedical fields [4–6]. In addition to carbon-based nanostructures, the significant silica content in rice husk offers another valuable component for nanomaterial synthesis and biomedical applications. Additionally, RH silica, owing to its biocompatibility and tunable surface properties, is widely explored for drug delivery applications, where its porous structure enables high drug loading and controlled release through surface chemistry or pore size modification[7]. Thus, RH represents a valuable precursor for advanced carbon- and silica-based nanomaterials with significant scientific, industrial, and biomedical importance.

Graphene oxide (GO), a derivative of graphene, consists of sp^2^-hybridized carbon atoms arranged in a hexagonal lattice but decorated with oxygen-containing functional groups. These groups enhance its dispersibility in aqueous media and provide sites for chemical modification, making GO highly versatile. Derived from carbon-rich wastes such as agricultural residues and plastics, GO offers an economical and environmentally friendly route for developing advanced nanomaterials [8–15]. Its unique biological, mechanical, and chemical properties have enabled applications in water purification, energy storage, bio-imaging, polymer development, drug delivery, and antimicrobial treatments. Incorporation of silver nanoparticles (AgNPs) into GO further enhances its biomedical relevance, as AgNPs are well known for their strong antimicrobial and cytotoxic effects. Several studies have demonstrated that GO/Ag nanocomposites exhibit remarkable antibacterial activity against pathogens such as *Escherichia coli* and *Staphylococcus aureus* [16], as well as cytotoxicity against multiple cancer cell lines, likely through mechanisms involving apoptosis and interference with cellular functions [17].The oxygen-containing functional groups of GO, including hydroxyl, epoxide, and carboxyl groups, confer excellent aqueous dispersibility and biocompatibility, pivotal for biomedical application as drug carriers. GO’s large surface area supports high drug loading efficiency through mechanisms such as π-π stacking and hydrogen bonding, making it suitable for targeted delivery of anticancer drugs. Functionalization with metal nanoparticles such as silver enhances the antibacterial and anticancer properties of GO-based nanomaterials, making them multifunctional platforms for cancer therapy. Furthermore, the sustainable synthesis of GO from biomass like rice husks aligns with green chemistry principles, offering environmental benefits without compromising biological efficacy [18–20].

Al-Assaly et al. developed an eco-friendly approach for synthesizing rGO/AgNP nanocomposites. The resulting material exhibited Notable antimicrobial effectiveness, against *Escherichia coli* and *Staphylococcus aureus*, highlighting its promising applications in the biomedical field [16].Ramadan et al. Conducted research on the environmentally friendly production of GO/Ag nanocomposites, chitosan serves as both a reducing and stabilizing agent.The synthesized nanocomposites displayed notable cytotoxic effects against human lung (H460), colon (HCT116), breast (MDA-MB-468), and hypopharyngeal (FaDu) cancer cell lines. These findings indicate that GO/Ag nanocomposites hold promise as potent anticancer agents, likely through mechanisms such as apoptosis induction and interference with cellular functions [17]. This work explores a novel AgNO_3_ functionalized GO derived from rice husk, assessing its effectiveness as a nanocomposite in cancer therapy. This approach offers an environmentally responsible option for producing graphene-based materials for advanced medical applications. The RH-GO/AgNO_3_ nanocomposite is expected to exhibit enhanced cell targeting, controlled release, and selective cytotoxicity against cancer cells, thus minimizing adverse effects on healthy cells. This study seeks to contribute to developing efficient, biocompatible, and environmentally sustainable nanomaterials in oncology biomedical research.

## Materials and methods

Rice husk was obtained from agricultural land in nainitaluttarakhand, India, 0.2 µm nylon membrane filter, DD Water (DDW), Ethanol, Silver nitrate (AgNO_3_, ≥ 99% purity), 5-Fluorouracil (5-FU, ≥ 98% purity) and sodium citrate tribasic dihydrate (Na_3_C_6_H_5_O_7_·2H_2_O) were obtained from Sigma Aldrich. DMEM (Dulbecco's Modified Eagle Medium, AT149-1L) from Himedia, supplemented with 10% FBS (Fetal Bovine Serum, RM 10432) from Himedia and 1% antibiotic solution (Penicillin–Streptomycin, P0781) from Sigma-Aldrich. FITC Annexin V, Propidium Iodide (PI) was a conventional, Pre-prepared nucleic acid dye, 10X Annexin V Binding Buffer, 0.1 M Hepes/NaOH (pH 7.4), 1.4 M NaCl, and 25 mM CaCl_2_ were purchased from Elabscience.

Fourier Transform Infrared (FTIR) spectroscopy measurements were conducted using a PerkinElmer Instrument (model: Spectrum Two)spectrometer equipped with an attenuated total reflectance (ATR) accessory. Samples of RH-GO and RH-GO/AgNO_3_ were prepared by the potassium bromide (KBr) pellet method: approximately 1–2 mg of finely ground sample was homogeneously mixed with about 100–200 mg of dry KBr powder, which is transparent to infrared radiation. The mixture was then placed in a pellet die and compressed under high pressure using a hydraulic pellet press to form a clear, thin pellet suitable for FTIR analysis. Spectra were recorded in the range of 400–4000 cm⁻^1^ with a spectral resolution of 4 cm⁻^1^, averaging 32 scans per sample. Baseline correction and normalization procedures were applied using the instrument software to enhance spectral quality. These parameters ensured high signal-to-noise ratio and reproducibility of the recorded spectra.

### Synthesis of rice husk derived GO (RH-GO)

Rice husk-derived graphene oxide (RH-GO) sheets were synthesized using a solvothermal approach, employing ethanol and double-distilled water (DDW) as solvents. Initially, collected rice husks were spread in a clean, dry area to reduce moisture content. While dark drying is a conventional method, mechanical drying can also ensure consistent dryness. The husks were then sieved or subjected to air blowing to remove dust, dirt, and other foreign particles.

For surface water removal, 100 g of brown rice husk was ground using a mixer grinder and mixed with 400 mL of DDW to form a uniform paste. This paste was combined with ethanol in a 1:1 ratio to prepare the solvothermal reaction mixture. The slurry was transferred to a Teflon-lined autoclave and heated at 110 °C for 36 h to yield a brown solid residue. The resulting solid was further crushed and dispersed in 30 mL of DDW, followed by magnetic stirring for 45 min. Coarse particles and contaminants were removed via centrifugation at 6000 rpm for 30 min at room temperature. The supernatant was subsequently filtered through a 0.2 µm nylon membrane to obtain a clear light-brown solution.

The filtered solution was concentrated to 100 mL and dried at 60 °C to yield approximately 50 mg of RH-GO, corresponding to a solution concentration of 1 mg/mL. To ensure reproducibility, the entire procedure was repeated four times. The final RH-GO product was ground using a mortar and pestle to obtain a fine powder suitable for storage and further experimentation [21].

### Functionalization of RH-GO with AgNO_3_

The synthesis of AgNO_3_wascarried out usingpreviously reported method [22].The synthesis of silver nitrate (AgNO_3_) nanoparticles and their functionalization with rice husk-derived graphene oxide (RH-GO) involve a series of chemical and physical processes. Initially, after dissolving AgNO_3_ in DDW, it is ultrasonically stirred for half an hour. To this solution, sodium citrate (Na_3_C_6_H_5_O_7_) is slowly added, operating as both a stabilizing and reducing compound. The citrate ions facilitate the reduction of Ag⁺ ions to metallic silver (Ag⁰), leading to the formation of silver nanoparticles (AgNPs). This reduction reaction results in a color change to yellow, indicating the successful synthesis of AgNPs. After cooling to ambient temperature, the solution is centrifuged for 30 min at 8000 rpm to get rid of any unreacted solutes.

**Scheme 1:**
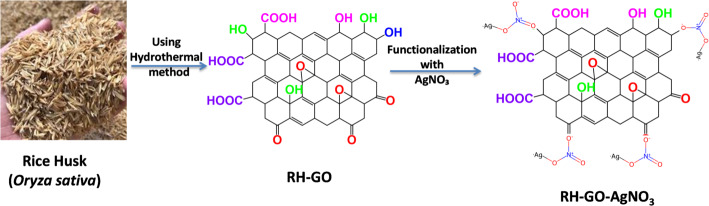
RH-GO functionalization with AgNO_3_

Discussing the process of functionalization, 2 mL of the colloidal AgNO_3_ solution is combined with 10 mg of RH-GO. The functional groups that include oxygen in RH-GO, like carboxyl, hydroxyl, and epoxy groups, encourage its AgNPs to bind via electrostatic interactions, π-π stacking, and covalent bonding. Sonication for 15 min ensures uniform dispersion and strong attachment of AgNPs onto the RH-GO surface. The final AgNO_3_-functionalized RH-GO material is then dried at ambient temperature, forming a stable nanocomposite suitable for various applications, including antimicrobial and catalytic purposes (Scheme 1).

## Drug loading

In short,4 mL of phosphate buffer solution (PBS, pH 7.4) with 1 mgRH-GO/AgNO_3_ composite was homogenized with 2 mL of 4 mg/mL 5FU solution in DMSO and stirred at ambient temperature for one day [23]. The drug-loaded nanocomposite was then separated by centrifugation and rinsed 4–5 times with deionized water. Finally, the purified drug-loaded nanocarrier was let to dry overnight at 40 °C in a vacuum oven.The following formula were used to calculate the drug loading capacity (LC%) and entrapment efficiency (EE%) of drug loaded composite. Folic acid functionalized graphene oxide nanosheets via plasma etching as a platform to combine NIR anticancer phototherapy and targeted drug delivery [24].

1 $$ {\text{LC}}\% = \frac{{\left( {{\text{Total amount of drug}}{-}{\text{Free amount of drug}}} \right)}}{{\text{Nanoparticles weight}}}*{1}00 $$

2 $$ {\text{EE}}\% = \left( {\frac{{\text{Initial amount of drug used}}}{{\text{Amount of drug encapsulated}}}} \right) \times {1}00 $$

### Drug release

To evaluate the drug release mechanism, 10 mg of 5-FU-loaded RH-GO/AgNO_3_ nanocomposite was dispersed in 30 mL of buffer solution (pH 4.0 and 7.4, tested in separate experiments). The beakers were maintained on a constant shaker at room temperature for three days. At predetermined time intervals, 2 mL of the supernatant was withdrawn and replaced with an equal volume of fresh buffer to maintain a constant solution volume throughout the experiment.

The maximum absorption wavelength (λ max) for 5-FU solutions was measured at 266 nm [25].

### MTT assay

For the in vitro cytotoxicity assay, HeLa cells were obtained from the National Centre for Cell Sciences (NCCS) in Pune, India. The cells were maintained in Dulbecco’s Modified Eagle Medium (DMEM) supplemented with 10% fetal bovine serum (FBS**)**and1% penicillin–streptomycin to support growth and prevent microbial contamination. They were incubated under optimal conditions in a humidified atmosphere containing 5% CO_2_ at 37 °C. To evaluate the cytotoxic effect of each sample, the half-maximal inhibitory concentration (IC_50_) was calculated, representing the concentration required to reduce cell viability by 50%.

### ROS measurement and cellular apoptosis

As previously reported, 2′,7′-dichlorofluorescein diacetate (DCFDA) was used in flow cytometry to assess reactive oxygen species (ROS) [23]. HeLa cells were cultured for 24 h at 37 °C and 5% CO_2_ after being plated at a density of 7000 cells/well in 6-well plates using 1 mL of DMEM supplemented with 10% FBS and 1% antibiotic solution (Penicillin–Streptomycin). After 30 min of DCFDA (2 µM) staining at 37 °C, the treated cells were assessed an hour later using a FACS flow cytometer. ROS generation was quantified based on the fluorescence intensity of the oxidized DCF, which reflects intracellular oxidative stress levels. A significant increase in fluorescence indicates elevated ROS production due to nanocomposite treatment. This oxidative stress is a known mechanism of apoptosis induction in cancer cells [26]. The results help in correlating ROS levels with cytotoxicity and apoptotic outcomes observed in treated HeLa cells. Hence, the DCFDA assay is a crucial component in understanding the mode of action of RH–GO/AgNO_3_ in inducing cell death.As previously reported, 2′,7′ dichlorofluorescein diacetate (DCFDA) was used in flow cytometry to assess reactive oxygen species (ROS) [27]. HeLa cells were cultured for 24 h at 37 °C and 5% CO_2_ after being plated at a density of 7000 cells/well in 6 well plates using 1 ml of DMEM supplemented with 10% FBS and 1% antibiotic solution (Penicillin–Streptomycin) [28]. After 30 min of DCFDA (2 µM) staining at 37 °C, the treated cells were assessed an hour later using a FACS flow cytometer.

Cellular apoptosis is a form of programmed cell death that eliminates cells without harming surrounding tissues, following a tightly regulated sequence of events. It plays a vital role in maintaining tissue homeostasis by balancing cell proliferation and death [29, 30]. In this study, HeLa cells were cultured and treated with various concentrations of the test samples. After treatment, the cells were washed twice with cold PBS and resuspended in 1X binding buffer at a concentration of 1 × 10^6^ cells/mL. The samples were then divided into four groups: Unstained cells, Control group, Annexin V only, and PI only. Annexin V-FITC and propidium iodide (PI) were added to the respective tubes as per the protocol. Each tube was gently vortexed, incubated at room temperature for 15 min, and then supplemented with 1X binding buffer. The stained cells were analyzed within one hour using a BD FACS Lyric™ flow cytometer, enabling the quantification of apoptotic and necrotic populations.

This dual-staining approach allows differentiation between viable, early apoptotic, late apoptotic, and necrotic cells based on membrane integrity and phosphatidylserine exposure. An increase in Annexin V-positive cells indicates apoptosis, while PI uptake reflects compromised membrane integrity associated with late apoptosis or necrosis. This method provides a sensitive and accurate evaluation of cell death mechanisms induced by the nanocomposite treatment.

### Statistical studies

Each experimental measurement in this study was conducted in triplicate. The mean ± standard deviation (S.D.) is used to display the findings. A two-tailed Student's t-test was used for statistical analysis, and a *p*-value of less than ≤ 0.05 was considered statistically significant. Excel and Microsoft Office were used for all statistical computations.

## Analysis and characterization

### RAMAN spectroscopy

**Fig. 1:**
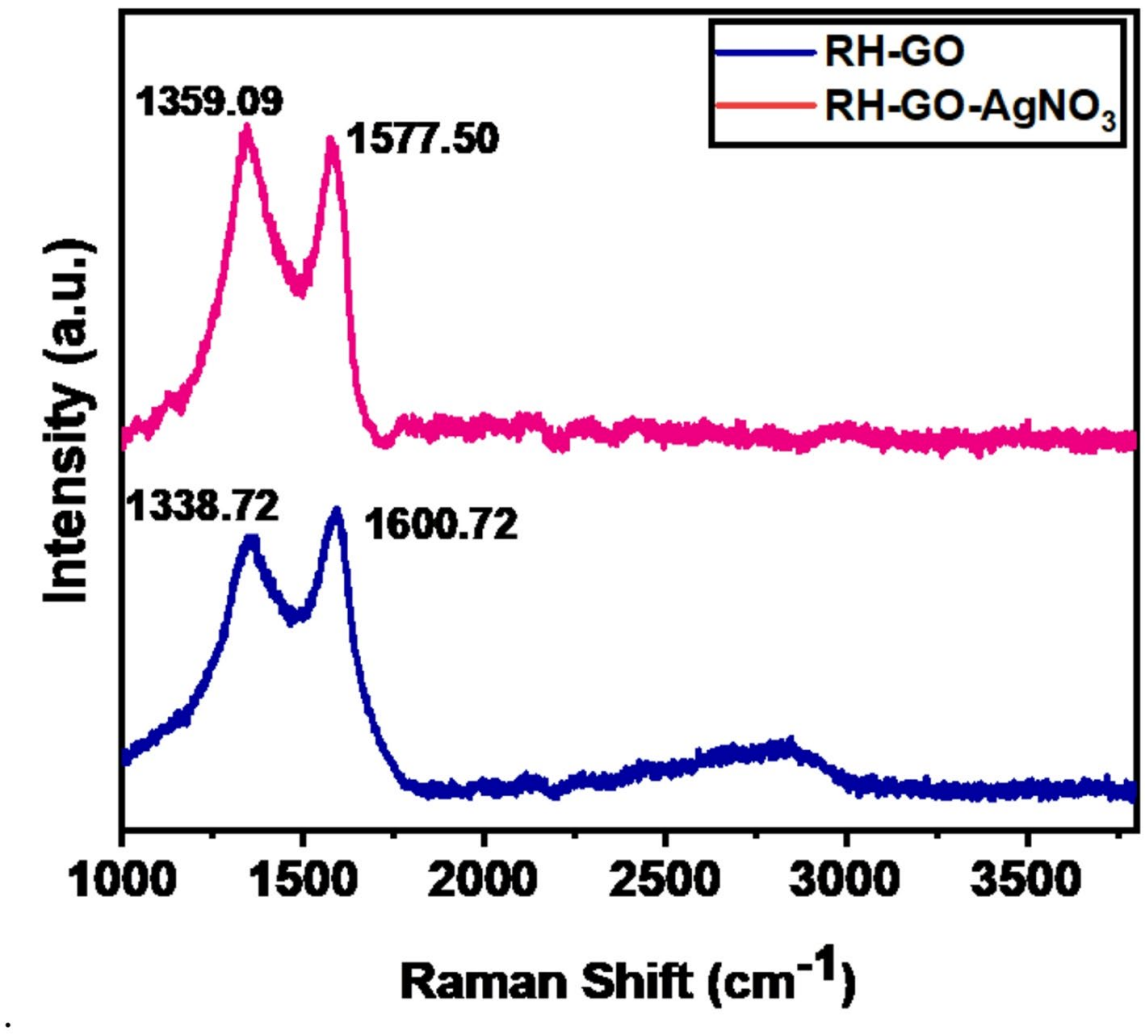
Raman spectrum of RH-GO and RH-GO/AgNO_3_

Raman spectroscopy is widely utilized to characterize various carbon materials [31, 32]. Figure 1 presents the Raman spectra of RH-GO and RH-GO/gNO_3_. The spectrum for RH-GO exhibits two prominent peaks the D-band at 1338.72 cm⁻^1^ and the G-band at 1600.72 cm⁻^1^. The D-band corresponds to the stretching vibrations in sp^3^ carbon atoms, which emerge due to the oxidation of graphite [33]. During oxidation, sp^2^ carbon atoms transition to sp^3^ carbon atoms in GO, causing these characteristic vibrations. The G-band, on the other hand, arises from the vibrations of sp^2^ carbon atoms, indicative of graphitic domains. The Raman spectra of RH-GO/AgNO_3_ exhibit two characteristic peaks: the D-band at 1359.09 cm⁻^1^, the G-band at 1577.75 cm. The degree of disorder in the graphene sheets can be evaluated from the intensity ratio of the D- to G-band (ID/IG). The observed ID/IG ratios for RH-GO and RH-GO/AgNO_3_ are 0.85 and 0.86, respectively, indicating a slight increase in structural disorder. This increase is attributed to the partial conversion of sp^2^ to sp^3^ carbon atoms on the GO surface upon adsorption of AgNO_3_, confirming successful functionalization. The increased ID/IG ratio, along with observed peak shifts, highlights the modification of RH-GO by AgNO_3_, enhancing its structural properties and potential applications.

### FT-IR analysis

**Fig. 2:**
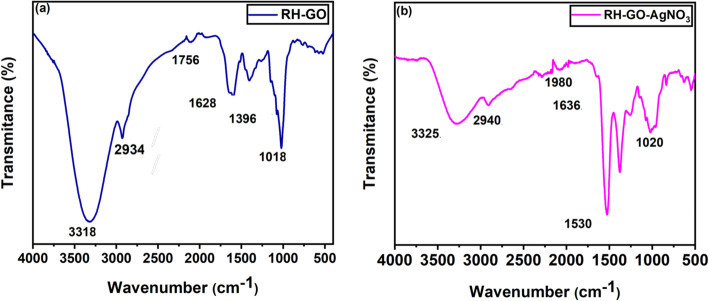
FT-IR analysis **a** RH-GO **b** RH-GO/AgNO_3_

An effective approach to identifying and analyzing functional groups in materials is Fourier Transform Infrared Spectroscopy (FT-IR). In this study, we analyzed rice husk-derived graphene oxide (GO) and silver nitrate-functionalized graphene oxide (RH-GO/AgNO_3_). FT-IR spectroscopy measures vibrations of molecular bonds that absorb infrared radiation at characteristic wavenumbers, revealing the presence of specific functional groups. Each functional group vibrates at specific frequencies influenced by atomic masses, bond strengths, and molecular geometry, resulting in distinct absorption bands. Figure 2 illustrates the FT-IR spectra of RH-GO and RH-GO/AgNO_3_ across the 400–4000 cm⁻^1^ range.In the spectrum of RH-GO, a broad absorption peak at approximately 3318 cm⁻^1^ corresponds to the O–H stretching vibration, indicating hydroxyl groups typically present due to surface oxidation and adsorbed water. The peak at 2934 cm⁻^1^ is attributed to the C–H stretching vibration in alkyl groups resulting from residual organics or reaction byproducts. The absorption bands at 1756 cm⁻^1^ and 1628 cm⁻^1^ correspond to C=O stretching in carboxyl groups and C=C stretching in aromatic rings, respectively, reflecting oxidized carbon domains and residual graphitic structures. Additional bands at 1396 cm⁻^1^ and 1018 cm⁻^1^ arise from C–O stretching in epoxy and alkoxy groups, further confirming the oxygenated functionalities on GO sheets[34].

For RH-GO/AgNO_3_, new features include a reduced intensity O–H stretching band near 3325 cm⁻^1^, indicative of interactions between silver ions and hydroxyl groups, potentially forming silver hydroxide species on the GO surface [35]. Shifts in the C=O stretching bands suggest coordination of the carboxyl groups with Ag⁺ ions, affirming successful functionalization. Additionally, there is an observed increase in intensity at the peak around 1530 cm⁻^1^. This band can be attributed to enhanced C=C stretching of the aromatic domains, and may also involve overlapping vibrations from N–O groups due to the presence of silver nitrate. The increased intensity is likely the result of increased conjugation or electron delocalization within the graphitic domains of GO upon functionalization, as well as possible interaction of silver nitrate with existing oxygen- or nitrogen-containing functional groups. Overall, changes in peak positions and intensities in RH-GO/AgNO_3_ spectra confirm the formation of a stable graphene oxide–silver nitrate nanocomposite with retained characteristic oxygenated groups from GO. These assignments align with standard FT-IR functional group identification, where C–O, C–C, C=O, C–H, and O–H groups show absorption in specific spectral regions, providing insight into material composition and chemical modifications.

### UV–vis spectroscopy

**Fig. 3:**
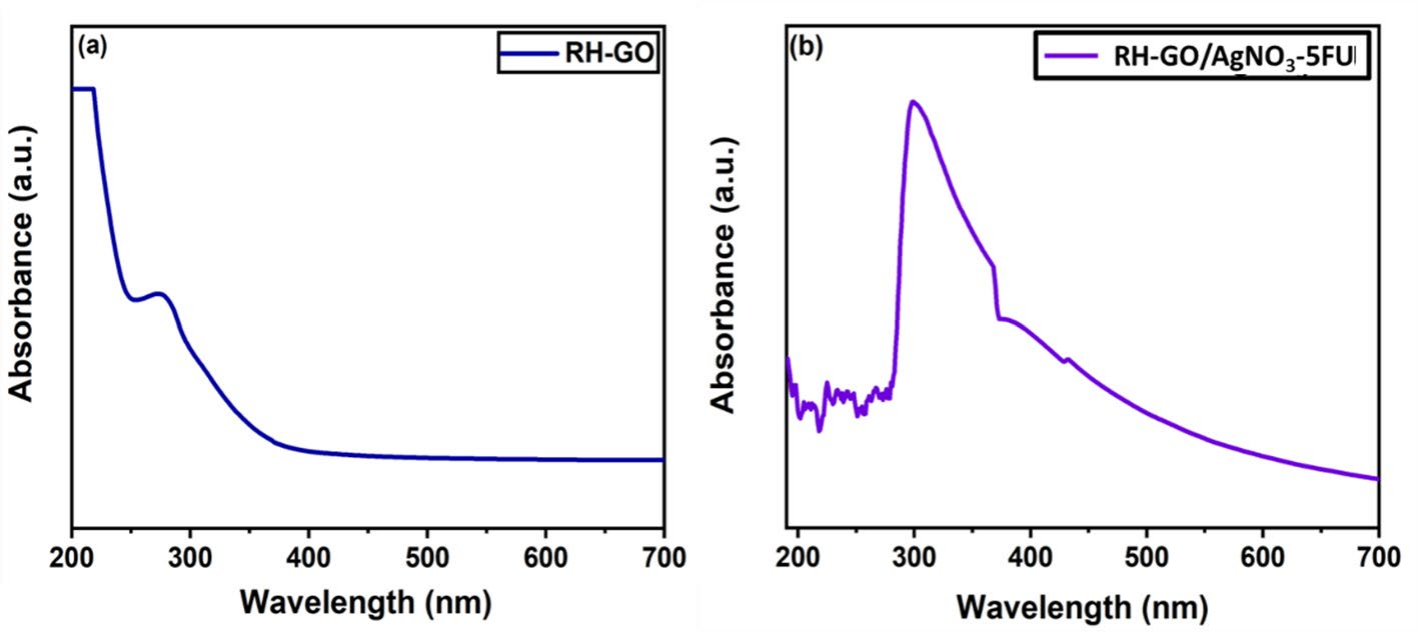
UV–Vis spectroscopy of **a** RH-GO **b** RH-GO-AgNO_3_-FU

UV–Vis spectroscopy serves as a highly effective technique for analysis, characterizing rice husk-derived graphene oxide and incredibly drug loaded RH-GO/AgNO_3_ composite. UV–Vis absorption graph shows wavelength on the x-axis 190–700 nm along the y-axis, and absorption. RH-GO and its composite with silver nitrate AgNO_3_ and 5FU typically show distinct absorption features. In Fig. 3 RH-GO, a strong absorption peak appears around 260 nm, indicating π=π transitions of the aromatic C=C bonds, whereas n=π transitions are represented by a shoulder at about 300 nm from oxygen-containing functional groups. Silver nanoparticles (AgNPs) were incorporated through the reduction of AgNO_3_ [36].

### Morphology analysis

**Fig. 4:**
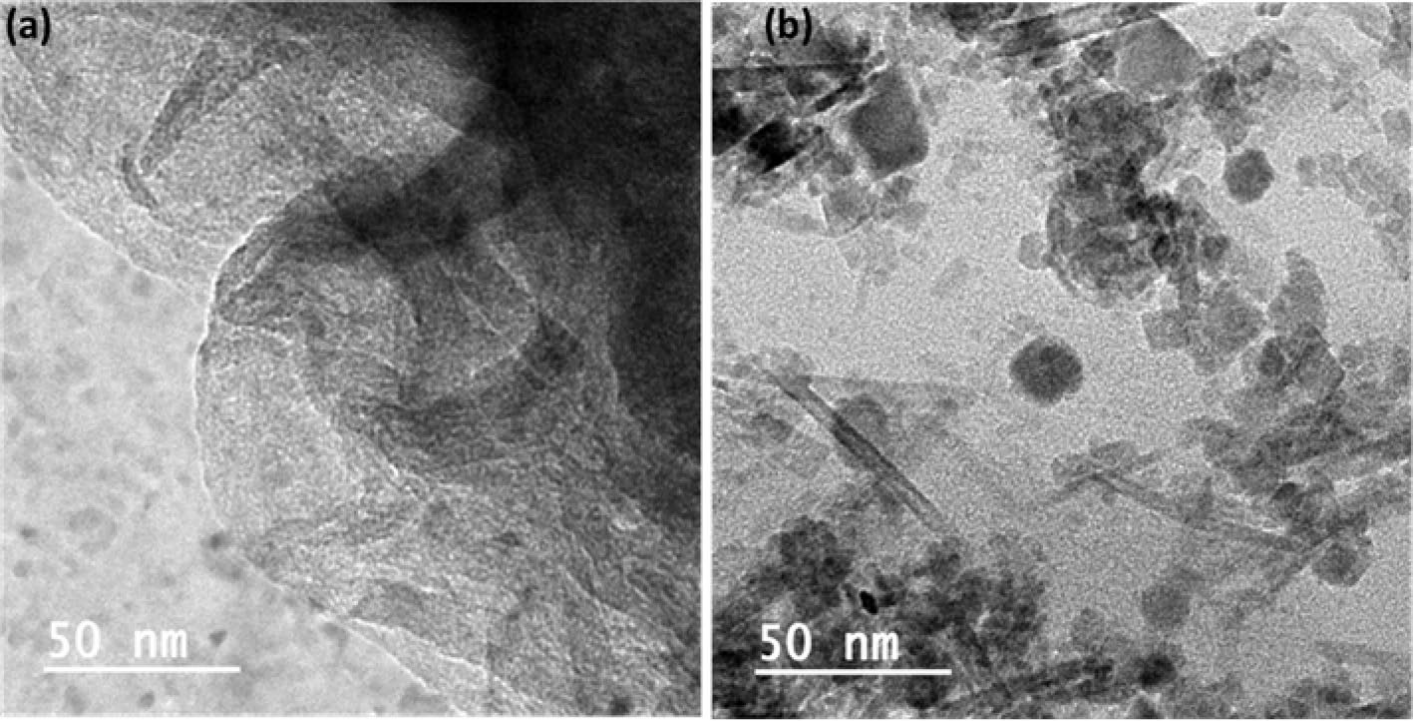
HR-TEM of **a** RH-GO **b** RH-GO-AgNO_3_

The morphological character was observed by the TEM study, which is analyzed in Fig. 4. The bare surface of RH-GO exhibits a stacked morphology (Fig. 4a). Following sonochemical reduction, the RH-GO/AgNO_3_ shows silver nanoparticles dispersed randomly across the RH-GO basal plane, as illustrated in Fig. 4b. Additionally, the agglomeration of silver nanoparticles on the graphene surface prevents the nanosheets from restacking, thereby enhancing electron transport properties. The morphology of the RH-GO and RH-GO/AgNO_3_ was examined by using HR-TEM. The image depicts the exfoliated RH-GO sheet, while RH-GO exhibits a lamellar structure with uniformly distributed, spherical nanomaterials embedded within the structure. RH-GO/AgNO_3_ observed in the TEM revealed successfully functionalized rice husk-derived GO functionalized with AgNO_3_ [37]. The surface morphology in bulk was analyzed using the SEM technique, as shown in Figs. S1a and S1b. The SEM pictures of RH-GO and RH-GO/AgNO_3_, reveals its amorphous and crumble structure in the bulk phase [38].

### Thermogravimetric TGA

**Fig. 5:**
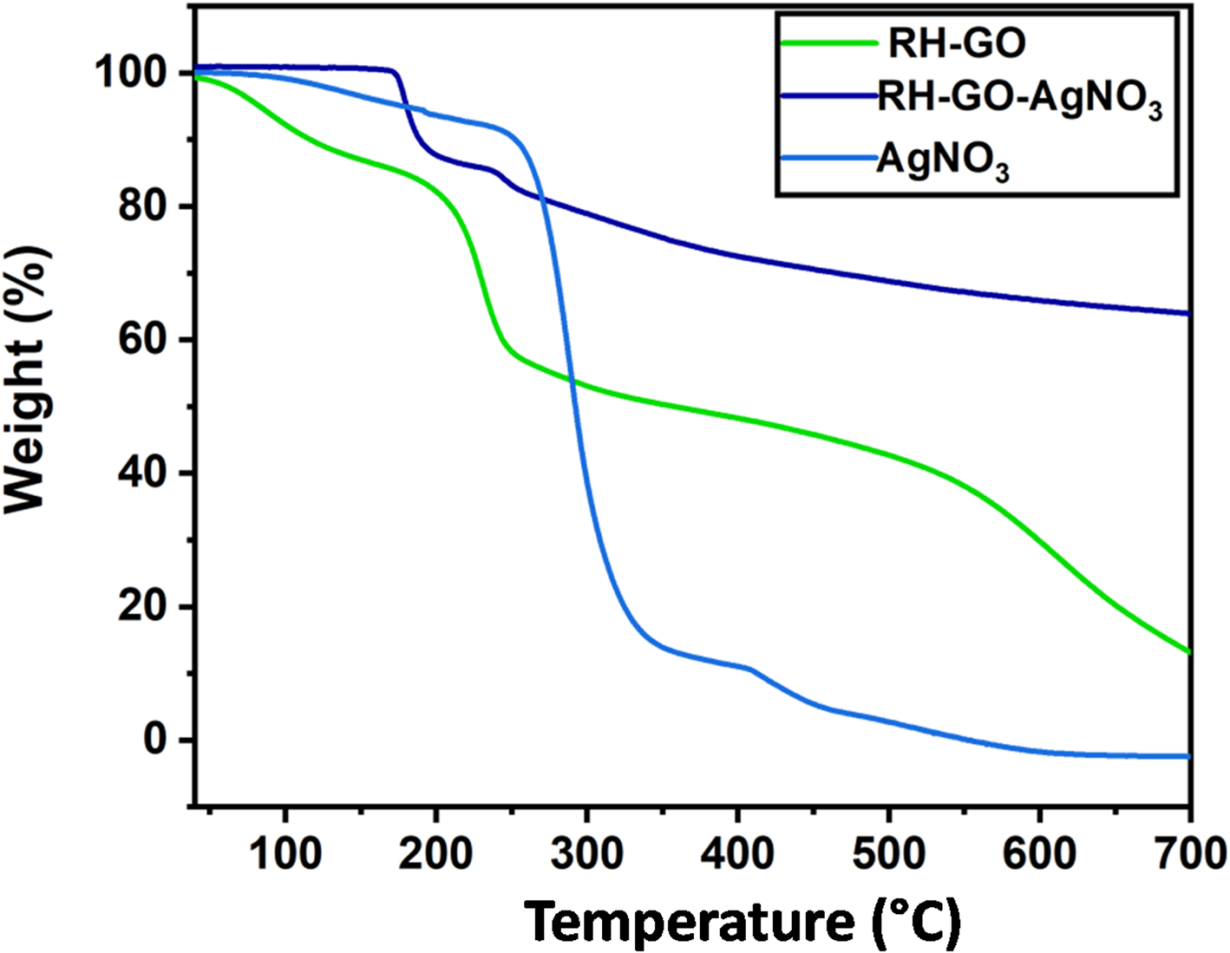
TGA graph of RH-GO, RH-GO/AgNO_3_, and AgNO_3_

TGA is a significant approach for evaluating the modifications in mass of a material over time or temperature under controlled conditions. The TGA spectra of RH-GO reveal that it is thermally unstable, exhibiting three stages of weight loss. Similar to RH-GO, the early reduction of weight below 100 °C is caused by the evaporation of water that has been absorbed. The second dropping of weight loss at approximately 195 °C results from deterioration of functional groups that are oxidative, while the third phase, occurring between 250 °C and 700 °C, is equivalent to the carbon structure being ignited. Notably, the RH-GO material retains approximately 20% residue at 700 °C, which is relatively high for a graphene-based material. This high residual mass is attributed to the significant silica content inherent in the rice husk precursor, which remains thermally stable at elevated temperatures and does not decompose during the TGA process. The presence of this inorganic silica phase contributes to the increased char yield and material stability, in addition to the carbonaceous structure of GO.TGA curve for RH-GO/AgNO_3_ shows a marked improvement in thermal stability following functionalization with AgNO_3_ [39]. At 700 °C, RH-GO retains 12.2% of its initial weight, while RH-GO/AgNO_3_ retains close to 64.8%. In contrast, pure AgNO_3_ shows nearly complete decomposition at this temperature (Fig. 5).This enhanced thermal stability in the synthesized nanocarrier RH-GO/AgNO_3_ can be attributed to the inorganic AgNO_3_ nanoparticles within the nanocomposite [40].

### X-ray photoelectron spectroscopy (XPS)

**Fig. 6:**
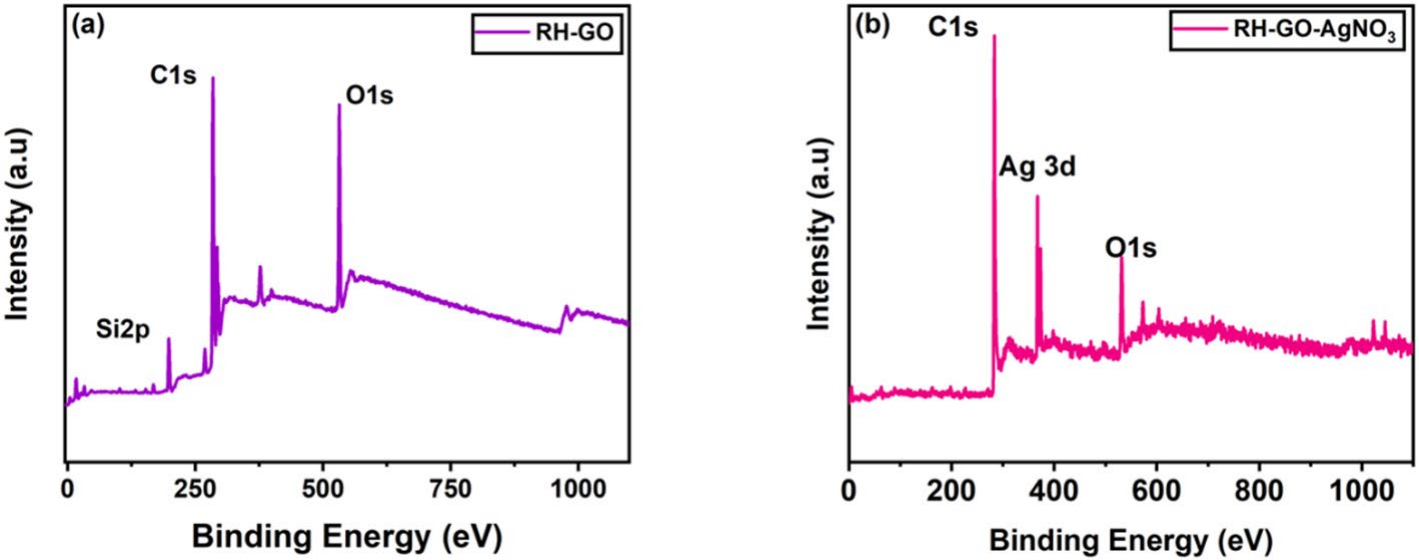
X-ray photoelectron spectroscopy (XPS) **a** RH-GO **b** RH-GO/AgNO_3_

XPS was completed for surface characterization of rice husk-derived RH-GO and Functionalized with AgNO_3_. Figure 5 displays entirety XPS spectrum of RH-GO having three peaks at 285.08, 201.24, and 532.19 eV ascribed to C1s, Cl 2P, and O1s, consequently. A peak at 201.24 eV to Ag 2p1/2, indicating silver species. In RH-GO functionalized with AgNO_3_, this suggests the presence of AgNO_3_ nanoparticles or residual silver ions, supported by the silver (Ag 3d), peak at ~ 199 eV. Peak at 398.08 eV in the context of RH-GO is typically attributed to pyridinic nitrogen (N). This form of nitrogen is incorporated during the nitrogen doping or functionalization of GO, where Two carbon atoms form a bond with N atoms within an aromatic ring. In RH-GO, this peak suggests that nitrogen has been successfully incorporated into the graphene oxide structure, improving characteristics including catalytic activity, surface reactivity, and electrical conductivity. The high-resolution spectra for Si2p, C1s and O1s yielded the binding energy values of RH-derived GO, confirming that carbon has three distinct chemical environments for GO produced from rice husks. Figure 6a shows C=C, C–C, C-H, and C–O at 199.12 eV, 285.08 eV, and 532.19 eV, respectively. Oxygen has two distinct chemical environments for RH-GO (ESI), according to binding energy values for O1s, which correspond to CeO at 529.9 eV and C–O at 530.8 eV. Figure 6b shows two distinct peak satellites that correspond to Si 2P3/2 at 285.08 and K2P1/2 at 285.08 eV [41]. The existence of functional groups (carbonyl, hydroxyl, and epoxy groups) on graphene nanosheets is confirmed by the XPS results [42].

Functionalization with AgNO_3_ reveals key features indicative of successful functionalization. The Ag 3d spectrum exhibits peaks at 367.50 eV confirming the presence of silver and distinguishing its ionic (Ag⁺) or metallic (Ag⁰) state through binding energy shifts. In Fig. 6b the C 1 s spectrum retains distinct peaks of pristine GO (C=C, C–O, C=O, O–C=O) but shows intensity changes or shifts due to interactions with Ag⁺ ions [43]. The O 1 s spectrum reflects modifications in oxygen-containing functionalities, evident through altered peak intensities or positions. Collectively confirming AgNO_3_'s efficient incorporation into the RH-GO matrix.

## Experimental details

### Drug loading

Utilizing UV–Vis spectroscopy, the loading of the 5FU into the RH-GO nanocomposite was examined [44]. For the first time, we used RH-GO-based silver nanocomposite (RH-GO-AgNO_3_) for enhancing poorly soluble 5FU stability and solubility. To verify the successful loading of 5FU the UV–Vis spectra of the nanocarrier of RH-GO/AgNO_3_ solutions were done, both with and without 5FU. For this, the drug was successfully loaded onto the nanocomposite by mixing a 5FU solution with an aqueous solution of RH-GO-AgNO_3_. Figure 3 represents the UV–vis spectrum of RH-GO and RH-GO/AgNO_3_-5FU. Due to the π to π* electronic transition of the C=C bond, the UV–vis spectrum of GO showed a strong distinctive absorption peak at 240 nm. The UV–vis spectrum RH-GO/AgNO_3_-5FU (Fig. 3b) displays an absorption peak of the 5FU drug molecule, It had been detected at about 266 nm with RH-GO/AgNO_3_-FU nanocomposite absorption peak [45].

**Fig. 7:**
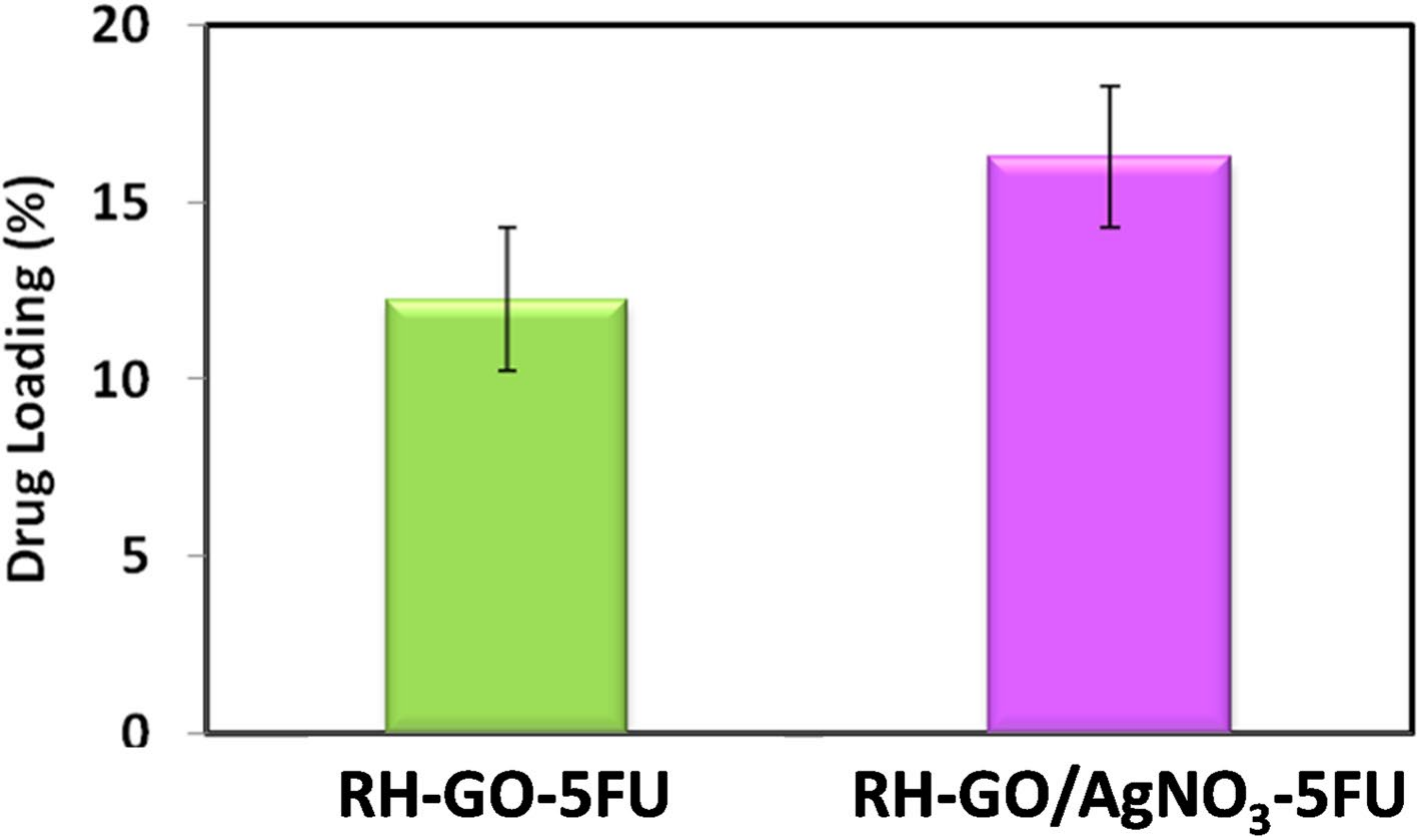
Drug loading capacity comparison of RH-GO-5FU and RH-GO/AgNO_3_-5FU

The loading capacities of 5-FU in RH-GO and RH-GO/AgNO_3_ attachment with AgNO_3_ nanocomposite were 12.4 ± 1.36% and 16.25 ± 1.42%, respectively. From the above results, it is observed that the capacity for loading drugs of RH-GO/AgNO_3_ is greater eventually the RH-GO (Fig. 7). The reason for this increased drug loading capacity is that 5FU interacts with RH-GO due to π–π interactions, hydrogen bonds, and hydrophobic interactions only. However, in the instance that RH-GO/AgNO_3_ composite, the drug is encapsulated within a hydrophobic inner cavity and also interacts with the terminal functional groups of the RH-GO/AgNO_3_ structure which helps in higher drug loading.

### Drug release

**Fig. 8:**
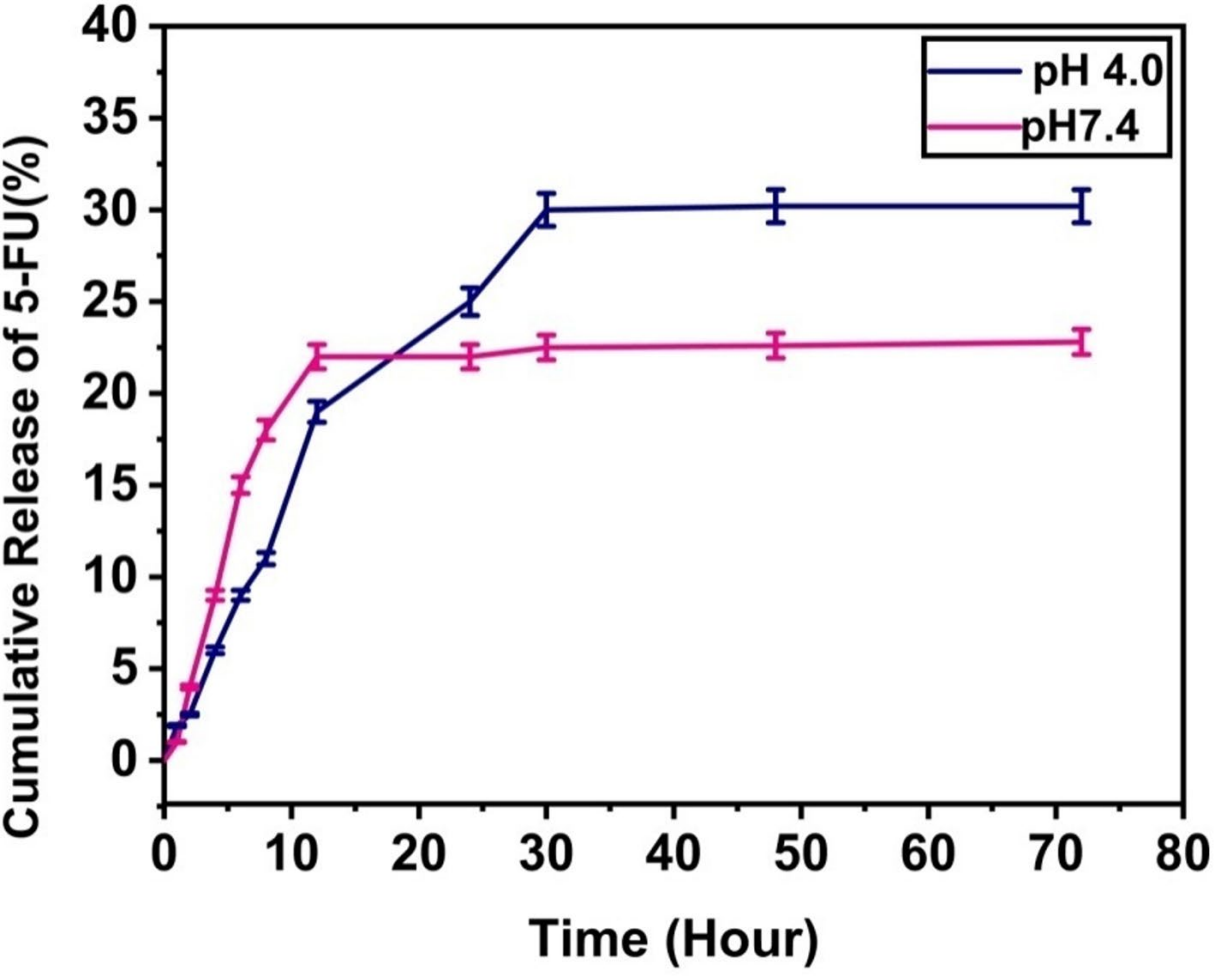
Release profile of 5FU from RH-GO/AgNO_3_-5FU

The drug release behavior of the nanocomposite was evaluated through *in-vitro* analysis under two pH conditions pH 7.4 and pH 4, as illustrated in Fig. 8. Under physiological conditions (pH 7.4), the release of 5FU from the RH-GO/AgNO_3_-5FU nanocomposite was relatively slow, with approximately 22.5% of the total bound drug released over 72 h. Conversely, at an acidic pH of 4, drug release was initially more rapid but slowed after 12 h. By the end of the 72-h period, 32.60% of the total 5FU had been released at pH 4. This demonstrates the pH-sensitive nature of the synthesized nanocomposite [45].

The slower drug release at pH 7.4 can be assigning to the drug and nanocomposite's strong hydrogen bond, significantly diminished in acidic conditions. Given that cancer cells often have acidic lysosomes, it is proposed that the RH–GO/AgNO_3_-5FU nanocarrier is internalized through endocytosis, enabling targeted drug delivery within the acidic lysosomal environment [45]. At pH levels below 5.4, the acidic conditions lead to protonation, which weakens the hydrogen bonds between the drug and the nanocarrier due to strengthened electrostatic repulsion, thereby promoting enhanced drug release.

### ROS estimation cellularapoptosis with flow cytometry-HeLa

**Fig. 9:**
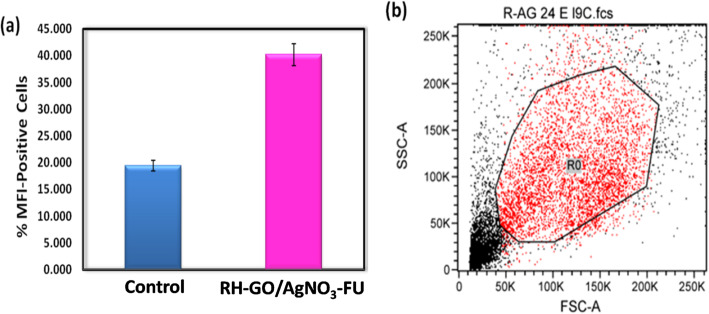
**a** ROS estimation HeLa cells **b** Positive Population Percentage

Developing a new nanocomposite capable of inducing apoptosis in cancer cells represents a promising strategy for cancer treatment. Graphene-based nanocomposites, such as RH–GO functionalized with AgNO_3_, have shown potential biomedical applications, including selective delivery and controlled release of anticancer drugs [46]. Figure 9 shows the flow cytometry analysis of ROS estimation in HeLa cells. Cells treated with the RH–GO/AgNO_3_ nanocomposite exhibited a significant increase in ROS generation compared to the control group. Specifically, the mean fluorescence intensity (MFI) of ROS-positive cells was markedly higher in RH–GO/AgNO_3_-treated cells (40.17%) compared to untreated control cells (19.45%). These findings indicate that the RH–GO/AgNO_3_ nanocomposite effectively enhances ROS production, which in turn contributes to mitochondrial dysfunction and apoptosis induction in HeLa cells.

### Cellular apoptosis with flow cytometry

**Fig. 10:**
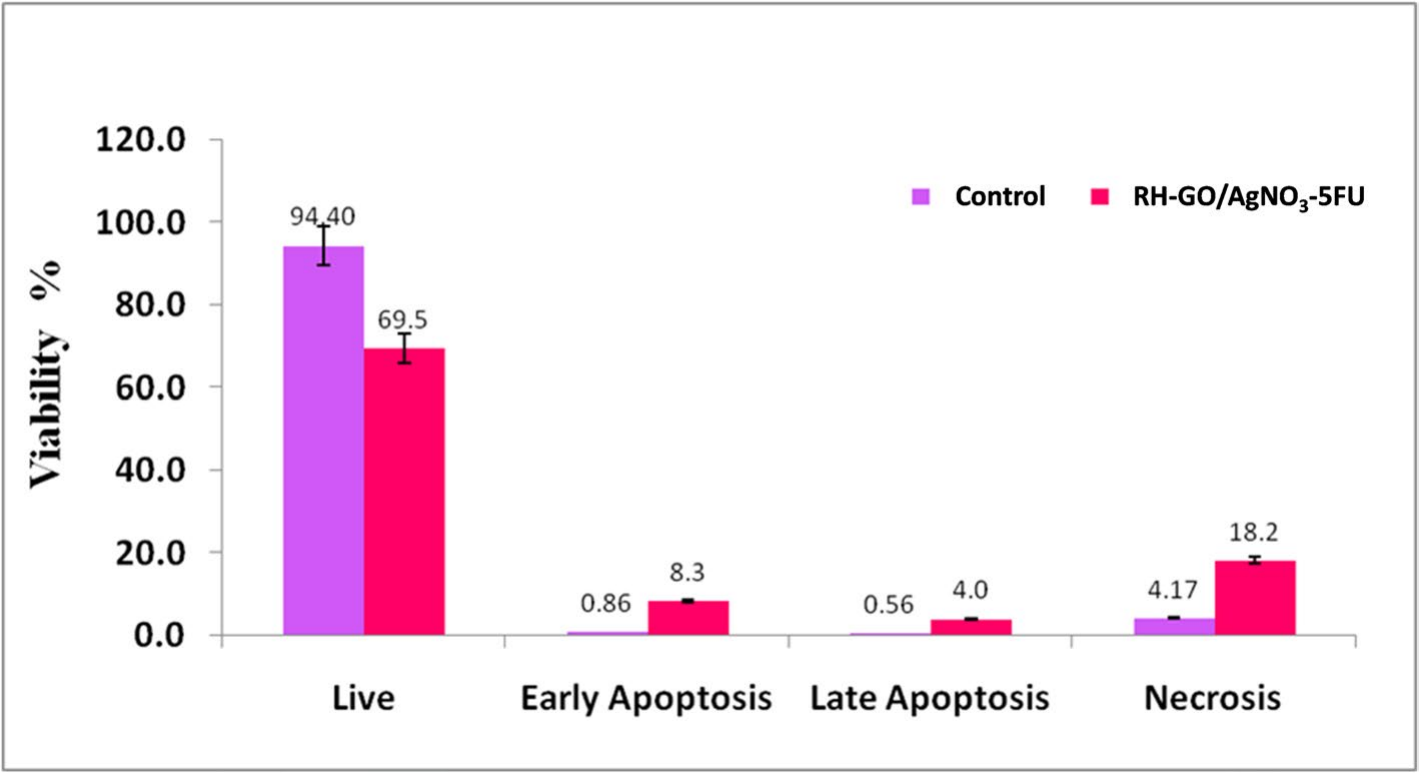
Cellular Apoptosis of RH-GO/AgNO_3_-FU in HeLa cells

Flow cytometry is utilized for analyzing cellular apoptosis, revealed distinct differences between the control group and the sample treated with RH–GO/AgNO_3_-5FU. In the control group, most cells (94.40%) remained viable, with only a small percentage undergoing apoptosis: 0.86% in the early apoptotic phase and 0.56% in the late apoptotic phase. Additionally, 4.17% cells were observed in the necrotic phase, indicating minimal cytotoxicity under normal conditions. In contrast, treatment with RH–GO/AgNO_3_-5FU significantly reduced cell viability to 69.5%, highlighting the cytotoxic effects of the nanomaterial (Fig. 10). Apoptosis was markedly elevated, with 8.3% of cells in the early apoptotic phase and 4.0% in the late apoptotic phase, indicating an increase in programmed cell death. Furthermore, necrosis was significantly higher in the treated sample, with 18.2% of cells transitioning to the necrotic phase. These results suggest that RH–GO/AgNO_3_-5FU induces cell death predominantly through apoptotic pathways, with a notable increase in necrotic cell death, reflecting the material's potential for targeting cancer cells.

### Anticancer activity

The enhanced performance of RH–GO/AgNO_3_-5FU compared to RH–GO-5FU alone can be attributed to the synergistic interaction between graphene oxide and silver nitrate. The incorporation of AgNO_3_ not only increases the number of active sites for drug binding but also improves the surface reactivity and functionalization potential of the nanocomposite, resulting in higher drug-loading efficiency [47, 48]. Additionally, silver ions contribute inherent cytotoxic and antimicrobial properties, which complement the anticancer activity of 5-FU, leading to improved therapeutic outcomes. The presence of Ag⁺ also facilitates better cellular uptake and promotes pH-responsive drug release in the acidic tumor microenvironment, enabling more targeted and effective drug delivery. Furthermore, AgNO_3_ enhances the structural and thermal stability of the nanocomposite, ensuring sustained drug release and improved biocompatibility, thereby making RH–GO/AgNO_3_-5FU a more effective nanocarrier than RH–GO-5FU alone.

### MTT assay

The cytotoxic effects of RH–GO, RH–GO/AgNO_3_, and RH–GO/AgNO_3_–5FU were evaluated *in-vitro* using the HeLa cell line [47]. Different quantities of these materials (10, 50, 100, 250, 500, and 1000 µg/mL) were tested. After a 24-h incubation period,MTT solution (5 mg/mL) was added to the cells and incubated for two hours (Fig. 10). The group serving as the control was developed out of untreated cells, while cells without MTT served as the Blank group.

**Fig. 11:**
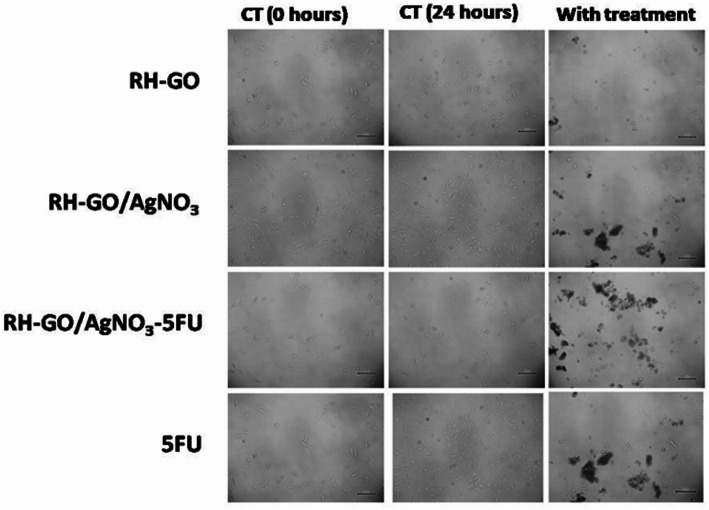
Representative images of HeLa cells by phase contrast microscopy

Throughout the time of incubation, the formazan crystals were dissolved in 100 µL of DMSO after the culture supernatant was withdrawn, and absorbance measurements were made using an ELISA plate reader at wavelengths of 540 and 660 nm. The 50% inhibitory concentration (IC50) was determined using GraphPad Prism 6 software and reported as the Mean ± SEM. Images of the cells were captured using an inverted microscope. Figure 11 illustrates the *in-vitro* viability of HeLa cells. With a concentration on of 1000 µgmL^−1^, the 5FU-loaded RH–GO/AgNO_3_ nanocarrier demonstrates significant cytotoxicity against HeLa cells, resulting in approximately 31% cell viability.

**Fig. 12:**
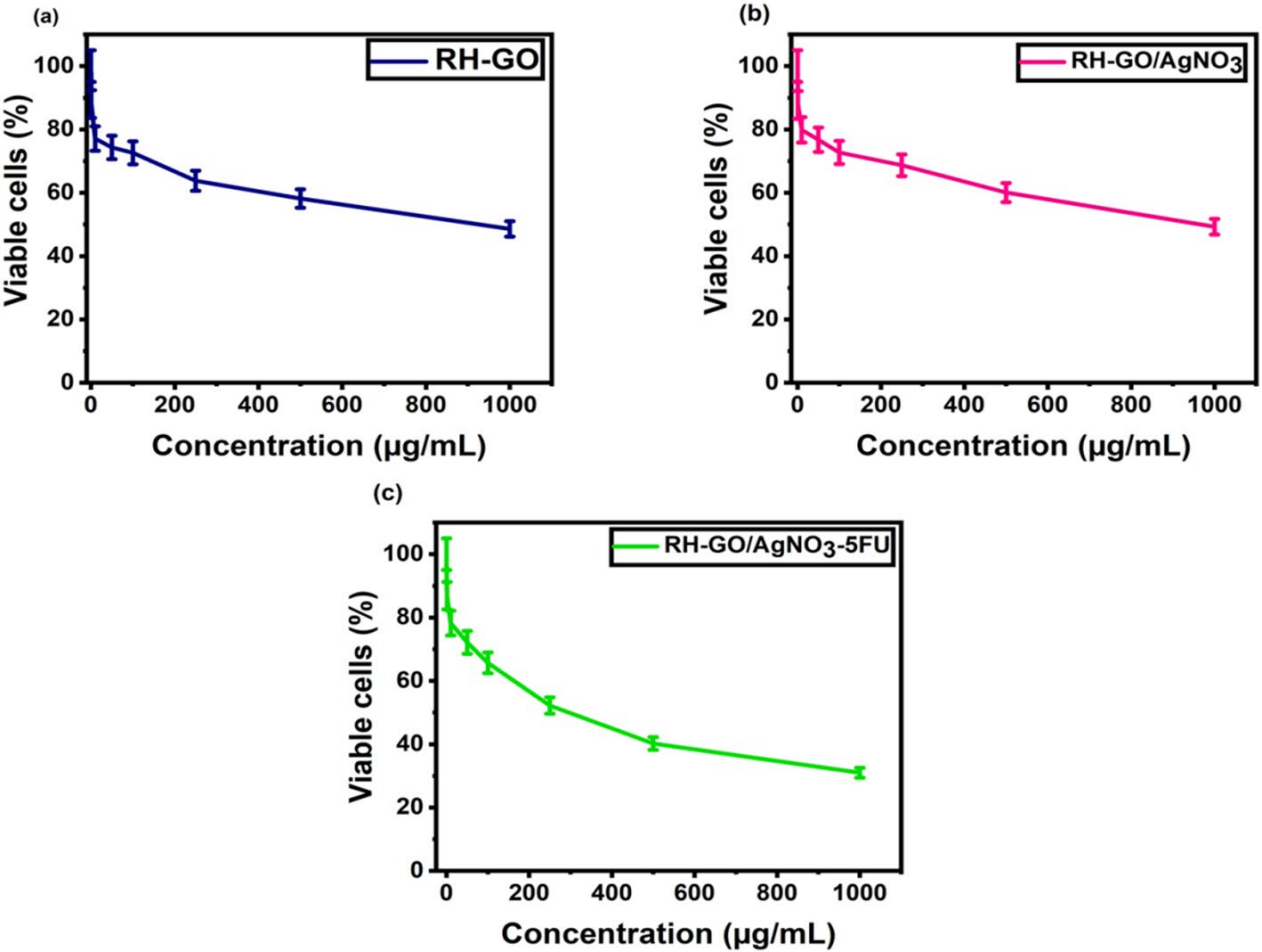
Cell viability MTT assay in different concentrations of RH-GO, RH-GO/AgNO_3_, and RH-GO/AgNO_3_-5FU

The final results of the MTT assay revealed the cytotoxic effects of the samples on HeLa cells at varying concentrations (Fig. 12). The values of the IC_50_, representing the Concentration necessary to inhibit 50% of cell viability, were established for every sample. RH-GO and RH-GO/AgNO_3_ exhibited IC50 values of 629.8 ± 0.13 µgmL^−1^, and 553.6 ± 0.14 µgmL^−1^respectively. In comparison, RH-GO showed lower cytotoxicity, with higher IC_50_ values, whereas the 5FU-loaded RH-GO/AgNO_3_ composite demonstrated the highest cytotoxicity, with an IC50 of 256.3 ± 0.11 µgmL^−1^, indicating that 5FU-loaded composite was the most potent.

## Conclusion

This study demonstrates the successful synthesis, characterization, and biomedical evaluation of rice RH-GO functionalized with AgNO_3_ for pH-responsive drug delivery. The functionalized nanocomposite showed enhanced thermal stability, effective drug-loading ability, and pH-sensitive release of the anticancer drug 5FU. The successful functionalization was validated by structural and morphological investigations, and *in-vitro* tests showed considerable cytotoxicity against HeLa cells, mostly via apoptotic mechanisms. When compared to its counterparts, the RH the purified drug-loaded nanocarrier was kept overnight at 40 °C in a vacuum oven to dry it RH–GO/AgNO_3_–5FU composite demonstrated higher anticancer efficacy, highlighting its promise as an environmentally benign, potent nanocarrier for targeted cancer therapy. Flow cytometry analysis indicated increased ROS generation and enhanced apoptosis in HeLa cells upon treatment with RH–GO/AgNO_3_-5FU, highlighting its cytotoxic effect via oxidative stress and programmed cell death. The composite induced both early and late apoptosis, along with elevated necrosis, indicating a multi-faceted cell death mechanism. This study exhibits the potential of using nanoparticles made from agricultural waste for cutting-edge biomedical applications. These findings the RH–GO/AgNO_3_-5FU nanocomposite shows promising potential as a smart, pH-sensitive, and biocompatible platform for targeted cancer therapy, warranting further investigation in preclinical models.

## Supplementary Information

Below is the link to the electronic supplementary material.

Supplementary Material 1.

## Data Availability

Data will be available on request.
