# Supplementary figures and images for: Silver nitrate functionalized rice husk-derived graphene oxide as a nanocarrier for pH-responsive drug delivery
Source: Discov Nano. 2025 Dec 20;20(1):237. doi: 10.1186/s11671-025-04400-w (PMC12718294; doi:10.1186/s11671-025-04400-w)

**Figure S1.** HR-TEM of (a) RH-GO (b) RH-GO-AgNO3.


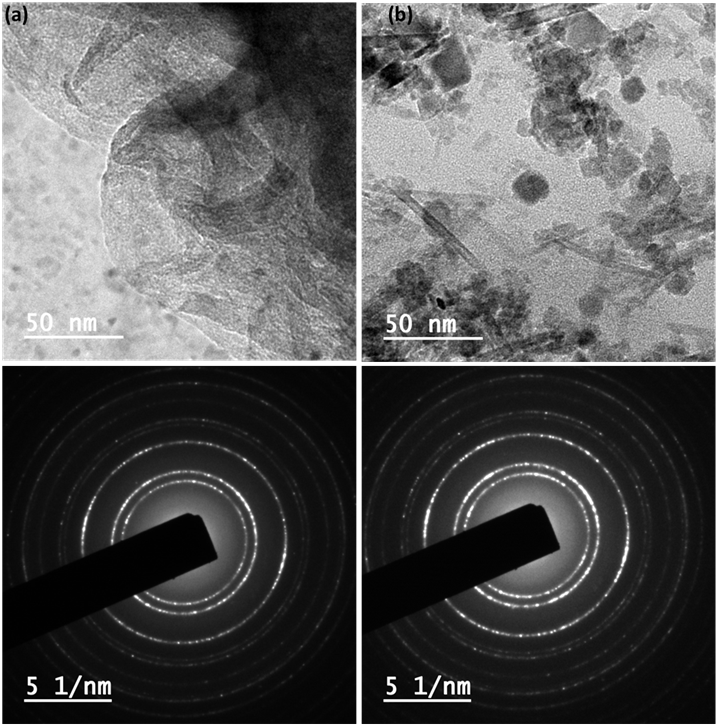


**Figure S2:** FE-SEM analysis (a) RH-GO (b) RH-GO/AgNO₃.

**
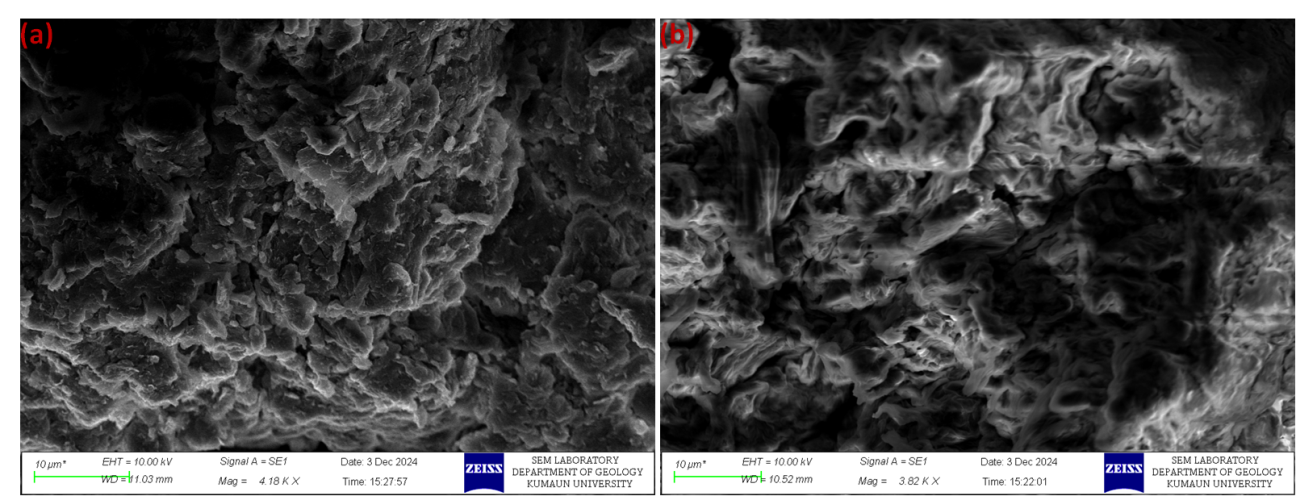
**

Supplement: Supplementary file 1 — Supplementary Material 1. [file 11671_2025_4400_MOESM1_ESM.docx]
